# Mitochondrial inhibition of uracil-DNA glycosylase is not mutagenic

**DOI:** 10.1186/1476-4598-3-32

**Published:** 2004-12-01

**Authors:** Sushant Kachhap, Keshav K Singh

**Affiliations:** 1 Sidney Kimmel Cancer Center, Johns Hopkins School of Medicine, Bunting-Blaustein Cancer Research Building, 1650 Orleans St., Baltimore, MD 21231 USA; 2 Department of Cancer Genetics, Cell and Virus Building, Room 247, Roswell Park Cancer Institute, Elm and Carlton Streets, Buffalo, NY 14263 USA

## Abstract

**Background:**

Uracil DNA glycosylase (UDG) plays a major role in repair of uracil formed due to deamination of cytosine. UDG in human cells is present in both the nucleus and mitochondrial compartments. Although, UDG's role in the nucleus is well established its role in mitochondria is less clear.

**Results:**

In order to identify UDG's role in the mitochondria we expressed UGI (uracil glycosylase inhibitor) a natural inhibitor of UDG in the mitochondria. Our studies suggest that inhibition of UDG by UGI in the mitochondria does not lead to either spontaneous or induced mutations in mtDNA. Our studies also suggest that UGI expression has no affect on cellular growth or cytochrome c-oxidase activity.

**Conclusions:**

These results suggest that human cell mitochondria contain alternatives glycosylase (s) that may function as back up DNA repair protein (s) that repair uracil in the mitochondria.

## Introduction

Mitochondrion plays an important role in various cellular functions ranging from synthesis of lipids to maintenance of ion homeostasis [1,2]. However, the singular function that defines this organelle is the production of energy by the electron transport chain. Mitochondrion is also a significant source of reactive oxygen species (ROS), known to be a potent DNA damaging agent [3]. The integrity of the mitochondrial genome is essential for effective cellular processes. The mitochondrion has various active and passive safe guard strategies to deal with the damaging effects of ROS on the mitochondrial DNA (mtDNA), one of them being the repair of the lesions caused by the ROS production [3]. Mitochondrial repair is not well studied. It is interesting to note that mtDNA experience more DNA damage than nuclear DNA [5]. Unlike the nuclear DNA that does not replicate in terminally differentiated cells mtDNA is continuously replicated in cells that have undergone differentiation. Hence lesions in the mtDNA can prove to be more deleterious [6]. Earlier it was believed that the mitochondria lack DNA repair mechanisms as thymidine dimers were not repaired in the mtDNA [4]. However, recent evidence indicates that DNA repair mechanism do function in the mitochondria [7,8,25,26]. Various enzymes that are involved in nuclear DNA repair have isoforms that are targeted to the mitochondria [9,10]. Whether these enzymes function in an identical fashion in the repair of both the nuclear and the mtDNA is not clear.

The uracil DNA glycosylase (UDG) removes misincorporated uracil or deaminated cytosine from DNA. Human UDG gene encodes two alternative spliced isoforms, UNG1 and UNG2 [11-13]. Of these the UNG1 is translocated to the mitochondria [14,15]. UNG2 localizes to the nucleus [15]. Although UNG2's role in repairing nuclear DNA is well established, the role for mitochondrial UNG1 is not well studied. In this paper we inactivated mitochondrial UNG1 by expressing a natural uracil DNA glycosylase inhibitor (UGI) from PBS2 phage that binds to the active site of UDG in equimolar ratio and inhibits the UDG enzyme [16]. UGI has been successfully used as a tool to examine the role of nuclear UNG2 in base excision repair of misincorporated uracil or deaminated cytosine in the nuclear DNA [17,18]. In order to elucidate the role of UDG in *in vivo *mtDNA repair we targeted UGI to the mitochondria to inhibit UDG activity. Our studies suggest that mitochondrial inhibition of UDG is not mutagenic. This study indicates that alternative DNA glycosylase(s) may be operative in the mitochondria that might repair uracil in the mitochondrial genome.

## Materials and Methods

### Constructs

The reading frame of uracil DNA glycosylase (UDG) that codes for functional UDG was amplified by PCR using forward primers (5'CCAGTGCCGCGCGCCAAGATCCATTCGTTGTTTGGAGAGAGCTGGAAGAAG) specific to human uracil DNA glycosylase that had a BssH II site at the 5' end and the reverse primers 5'TTGA TCTCGAGTCACAGCTCCTTCCAGTCAATGGG that had the Xho I site engineered at the 5' end. The template used for the amplification was pTUNGΔ84 [13]. The PCR fragment was cloned into pCMV/myc/mito (Invitrogen) treated with BssH II and Xho I. The vector has a mitochondial targeting signal of the subunit VIII of human cytochrome c oxidase that facilitates targeting of the cloned protein to the mitochondria. The construct was named as pCMV UNG.

The complete reading frame of uracil DNA glycosylase inhibitor gene was amplified using pTZUgi (a gift from Dr. Umesh Varshney) as a template with forward primers (5'CCAGTGCCGCGCGCCAAGATCC ATTCGTTGATGACAAA TTTATCTG ACATC) specific to uracil DNA glycosylase inhibitor from phage PBS2 that had a BssH II site at the 5' end and the reverse primer(5'CGCCCGTTTGATCTCGAGTTATAAC ATTTTAATCCATTAC) which had the Xho I site engineered at the 5' end. The PCR fragment was cloned into pCMV/myc/mito (Invitrogen). The construct was named as pCMV UGI.

### Transfections

Stable transfectants of the above constructs were made in immortalized normal breast epithelial MCF 12A cells using lipofectin as a transfecting agent. Briefly, MCF12A cells were plated to 70 % confluency in a 35 mm dish and transfected with 2 ug of pCMV UNG and pCMV Ugi. The cells were selected using G418 as a selection medium. The clones were selected after plating the cells in a 96 well plate to single cell dilution and the clones were screened for integration using PCR. A pCMV/myc/mito/GFP that has a GFP protein fused to the mitochondrial signal was used as a control to assay the efficiency of transfection and the expression of the protein using the vector. An empty vector was stably transfected and used as a control in all the experiments.

### PCR Screening of clones for stable integration of the constructs

Each construct was assayed for stable integration after transfection using PCR. The primers were the same that were used for amplifying the gene for cloning namely UDG specific primers, forward primer: 5'CCAGTGC CGCGCGCCAAGATCCATTC GTTGTTTGGAGAGAGCTGGAAGAAG reverse primer 5'TTGATCTCGAGTCAC AGCTCCTTCCAGTCAATGGG, for screening UDG stable integrants and UGI specific primers, forward primer 5'CCAGTGCCGCGCGCCAAGATCCATTCGTTGATGACA AATTTATCTGACATC and reverse primer 5'CGCCCGTTTGATCTCGAGTTATAAC ATTTTAATCCATTAC for screening Ugi stable integrants. Briefly, the each clone was transferred from the 96 well plate to a 24 well plate and DNA was extracted when the wells were confluent using standard methods. 100 ng of the DNA was used to PCR amplify the DNA that was transfected. Clones that showed an intact gene were selected for further analysis.

### Isolation of mitochondria

Stable clones and parental MCF12A cells were grown in T75 flask to seventy percent confluency. The cells were washed with 1X PBS and treated with 1.5 ml of 0.04% Digitonin solution (0.4 mg Digitonin /ml,2.5 mM EDTA,250 mM mannitol, 17 mM MOPS., pH 7.4). The cells were thoroughly resuspended and homogenized using ten strokes of a dounce homogenizer on ice. One ml of 2.5 X sucrose mannitol buffer (525 mM Mannitol, 175 mM Sucrose, 12.5 mM tris-HCl., pH 7.49) was added and homogenized further using 20 strokes of the homogenizer. Ten micro liter of the homogenate was visualized under the microscope to assess complete breakdown of the cells. The mitochondria were isolated by differential centrifugation [19]. The homogenate was centrifuged at 2500 rpm at 4°C to pellet the nuclei and the supernatant was further centrifuged at 2500 rpm till no pellet was visually observed. The supernatant was finally centrifuged at 14000 rpm at 4°C to pellet the mitochondria.

### Western Blotting

Stable transfectants were assayed for production of the UDG protein in the mitochondria by western blotting. Twenty micrograms of the mitochondrial protein was electrophoresd on a 12% SDS polyacrylamide gel and transferred on a nitrocellulose membrane. The membrane was blocked overnight in a blocking solution containing 5% non-fat milk and probed with the primary antibody (1:1000 dilution) against UDG (a gift from Dr. Hans Krokan, Norway). The membrane was washed twice with TBST and probed with a secondary antibody linked to horseradish peroxidase. The bands were visualized using ECL (Amersham Pharmacia) kit. The membrane was then probed for the house keeper protein beta actin to assess for equal loading.

### RT-PCR

RNA from Ugi stably transfected MCF 12A cells was extracted using TRIZOL reagent following the manufacturers instruction. One and a half micrograms of total RNA was used for reverse transcription using Superscript II Rnase H^-^reverse transcriptase (Invitrogen). Two microlitres of the reverse transcribed products was used in the subsequent PCR reactions. Twenty-five microlitres of the PCR reactions contained 20 mM Tris-HCL, pH 8.4, 50 mM KCl, 1.5 mM MgCl_2_, 200 μM dNTP and 10 picomoles of each primer (forward primer: 5'CCAGTGCCGCGCGCCAAGATCCATTCGTTGATGACAAATTTATCTGACATC and reverse primer 5'CGCCCG TTTGATCTCGAGT TATAACATTTTAATCCATTAC and one unit of Taq DNA polymerase (Invitrogen). The PCR profile consisted of an initial denaturation at 94°C for 5 minutes and 32 cycles of denaturation at 94°C for 45 sec, annealing at 58°C for 1 min and extension for 2 min at 72°C with a final extension at 72°C for 10 min. The PCR products were electrophoresed on a 1% agarose gel stained with ethidium bromide (0.5 μg/ml) and visualized under UV.

### Flow Cytometric Analysis

Proliferation assay was done using a flourescent lipophilic molecule, 5-(and-6)-carboxyfluorescein diacetate succinimidyl ester (CFSE) that gets incorporated into live cells and gets diluted into daughter cells with every cell division. The dilution in the intensity of the dye as estimated by flow cytometry with respect to a "0" hour time point gives an indication of the proliferation of the cells. Cells were plated at a density of 1 × 10^5 ^in a 60 mm dish and stained for 15 min using the fluorescent dye CFSE (Molecular Probes). Cells were fixed in 70% alcohol just after staining to have a 0 hour time point and after a period of 72 hours. Proliferation was then estimated using flow cytometry using a FACSvantage™, Becton Dickinson [20,21].

### SIN1-1 and SNAP treatment and of mitochondrial damage

MCF12A parental cells were used for dose optimization of the SIN1 and SNAP. An optimal dose was used for further experiments. The parental and the transfected cells were plated on a 60 mm dish to 70% confluency. Each of the cell lines were treated with 4 mM 3-morpholinosydnonimine (SIN-1) and 2 mM **S**-nitroso-N-acetylpenicillamine (SNAP), NO donors for a period of 1 hour after which the medium was changed and cells were harvested after 0, 2, 4, 6 hour period intervals. DNA was extracted from these cell lines and Cox I was PCR amplified and sequenced using an automated sequencer (ABI PRISM) for mutation analysis.

### Uracil DNA Repair Assay

Uracil DNA repair assay was conducted as described by Radany et al., [17]. Oligonucleotides used for the assay were and T-34-mer 5'AGCTTGGCTGCAGGTXGACGGATCCC CGGGAATT-3' containing a uracil or thymine residue at position 16 (X=U or T, respectively) and (A-34-mer and G-34-mer) 5'-AATTCCCGGGGATCCGTCXACCTGC AGCCAAGCT-3' containing an adenine or guanine residue at position 19 (X = A or G, respectively). Twenty picomoles of oligonucleotide substrates were labeled with ^32^P using T4 polynucleotide kinase. The labeled products were precipitated and then resuspended in a lower volume of distilled water. These were directly used as single stranded substrates in the enzyme assay. To prepare double stranded substrates twenty picomoles of the labeled products were annealed to 10 pmoles of the unlabelled complementary or mismatch oligos by heating at 70°C and slowly cooling it down to room temperature for an hour. UDG assay was performed using 50 μg of mitochondrial extract in 1X UNG buffer (20 mM Tris-HCl pH8.0,1 mM EDTA,1 mM DTT) and 4 pmoles of labeled oligos. The reaction was carried out at 30°C for 45 min. The assay using commercially available Ugi (NEB) was performed using similar conditions. Ten units of Ugi per reaction was used. Apyrimidinic sites (AP-sites) generated by uracil removal from DNA substrates were hydrolyzed by the addition 0.1 N NaOH and incubating for 10 min at room temperature and terminated using a formamide buffer (80% formamide in 1XTBE) to generate single stranded products. Half of the reaction was electrophoresed using a 15% acrylamide gel containing 8.3 M urea and 1X TBE buffer. The gel was autoradiographed after electrophoresis to visualize the bands.

## Results

### Generation of stable transfectants expressing UGI and UDG in the mitochondria

**Figure 1:**
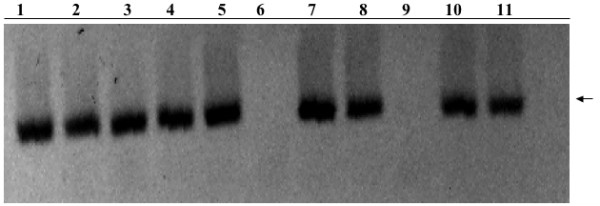
PCR screening for stable integrants of pTZUGI in MCF12A cells. PCR using pTZUGI primers were used to screen for stable integrants. Lane 1 is a positive control (pTZUgi plasmid DNA), lane 2, 3, 4, 5, 7, 8, 9 and 10 show the presence of stable integrants.

**Figure 2:**
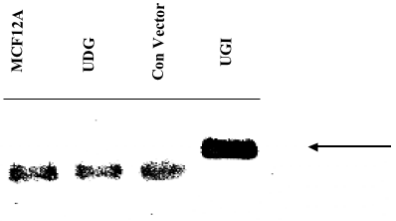
RT PCR to verify expression of Ugi gene transfected in MCF12A cells using primers specic to the UGI gene: RT PCR products electrophoresed on a 1% agarose gel. Lane 1 shows RT PCR product from MCF12A cell line, lane 2 shows RT PCR product from MCF12A cells transfected with pCMV UNG, lane 3 shows RT PCR product from MCF12A transfected with empty pCMV/myc/mito control vector, lane 4 shows RT PCR product from MCF12A transfected with pCMV UGI vector.

**Figure 3:**
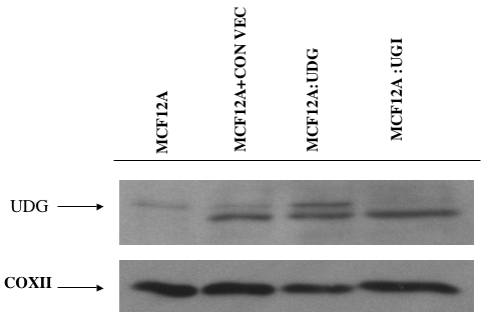
Western blot analysis of mitochondrial UDG expression in transfected cell lines: Upper panel shows western blotting of mitochondrial extracts with UDG antibody the lower panel shows the same blot probed with Cox II antibody to assess for equal loading of the samples. Lane 1 is mitochondrial extract from parental MCF12A cells, lane 2 is mitochondrial extract from MCF12A cells transfected with empty pCMV/myc/mito vector, lane 3 is mitochondrial extract from MCF12A cells transfected with pCMV UNG vector, lane 4 is mitochondrial extract from MCF12A cells transfected with pCMV UGI vector. A band of lower molecular weight was seen in some extracts.

Previous studies have shown that uracil DNA glycolyase can be inhibited by PBS2 phage protein UGI in a stoichiometric fashion [17,18]. This protein has been used to inactivate nuclear UDG by targeting it specifically to the nucleus by attaching a nuclear localization signal [17]. We have used the pCMV/myc/mito (Invitrogen) vector to target UGI protein in the mitochondria to inhibit UDG activity. Expression of UDG (UNGΔ84), that retains the wild type function of the enzyme, was also targeted to the mitochondria and was used as control. The pCMV/myc/mito vector contains a mitochondrial localization signal (MLS) of subunit VIII of human cytochrome c oxidase that specifically targets a protein of choice to the mitochondria. Clones containing stable integration were isolated and were confirmed by PCR upon transfection with UGI gene and the UDG after G418 selection. To confirm that the UGI gene was expressed in transfected cell lines we did RT-PCR analysis (Figure 1). Our results show that UGI was expressed (Figure 2). Western blot analysis on extracts isolated from mitochondria using antibody against UDG protein demonstrates that cells containing UNG stable integration express higher level of UDG protein in the mitochondria (Figure 3, lane 3). It is important to note that the UDG band was absent in cells expressing UGI because UDG epitope was not available for binding with antibody.

### Expression of UDG and UGI in the mitochondria does not affect cell growth

**Figure 4:**
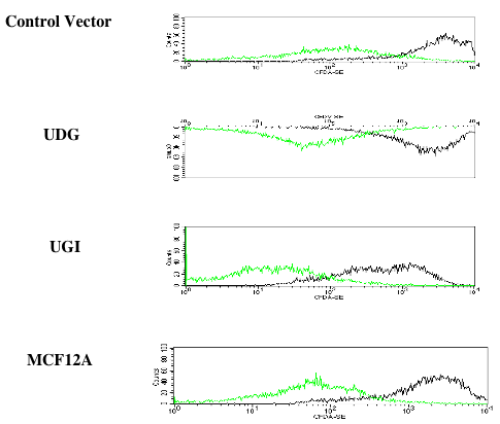
FACS analysis of growth rate using fluorescent dye CFDA-SE: The first graph (green) in each panel shows fluorescent cells at 0 hour time point and the second (black) shows a decrease in fluorescence at 72 hr after the cells proliferate. There is no difference in the growth rate between the parental cell line and the transfected one.

It is possible that inhibition of UDG in the mitochondria may affect cell growth. To determine if UGI expression in the MCF12A cells resulted in a difference in cellular growth, cell cycle analysis was conducted using flow cytometry. The cell cycle distribution of parental MCF12A cells, wild type UNG and UGI transfected cell line and the cell line containing the control vector is shown in figure 4. Interestingly, a very similar growth pattern was observed between all the cell lines examined. We conclude that expression of UGI in the mitochondria does not affect cell growth.

### Lack of mutations in COXI, COXII, and COXIII gene encoded by mtDNA

Our previous studies suggest that inactivation of UDG in yeast Saccharomyces cerevisae leads to mutations in mtDNA [25]. We therefore asked whether UGI transfected cells showed spontaneous increase in level of mutation in mtDNA. We isolated mtDNA from cell expressing wild type UNG, UGI and the control MCF12 A cells containing vector. We amplified mtDNA encoding COXI, COXII and COXIII by PCR. PCR fragments were sequenced. Sequencing revealed no differences in mtDNA sequence between the cell lines expressing UGI, wild type UNG1 and the cell line containing the vector (data not shown). We also treated the transfected cell lines with two agents SIN1 and SNAP. Both SIN1 and SNAP are known to deaminate mtDNA [22]. The transfected cells were treated for one hour with the agent and were harvested at different time intervals to accumulate mutations. The DNA from these cell lines was isolated and analyzed by sequencing for mutations in the COXI, COX II and the COX III genes encoded by the mtDNA. Our analysis showed no increase in mutation in mtDNA in the treated cell lines (data not shown). We conclude that UGI expression in the mitochondria does not lead to mutations in mtDNA.

### Uracil repair is unaffected by inhibition of UDG in the mitochondria

**Figure 5:**
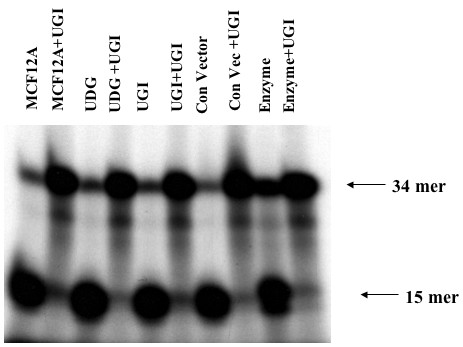
UDG activity in mitochondrial extracts of parental MCF12A cells and transfected cell lines: Lanes 1, 3, 5 and 7 show UDG activity in mitochondrial extracts from MCF12A parental cell line, cells transfected with pCMV UNG, cells transfected with pCMV UGI, cells transfected with pCMV/myc/mito control vector and commercially available UDG enzyme, that acted as a positive control, respectively. Lanes 2, 4, 6, 8 and 10 shows an inhibition of UDG activity when commercially available Ugi was added in mitochondrial extracts from MCF12A parental cell line, cells transfected with pCMV UNG, cells transfected with pCMV UGI, cells transfected with pCMV/myc/mito control vector and commercially available UDG enzyme, that acted as a positive control, respectively.

It has been previously reported that the UGI protein when targeted to the nucleus lowers the activity of the nuclear UDG enzyme [16]. To analyze the effect of UGI expression on the mitochondrial UDG activity in the transfected cell line, we carried out UDG activity measurements in mitochondrial extracts with and without commercially available UGI as a control. The commercially available UGI was found to inhibit mitochondrial UDG. However, constitutively expression of UGI in the mitochondria in cell line transfected with UGI was not observed (Figure 5). These results suggest two possibilities i) that an alternative uracil glycosyalase activity is present in the mitochondria and/or ii) mitochondrially expressed UGI is incapable of inhibiting UDG present in the mitochondria. Since commercially available UGI does inhibit mitochondrial UDG activity, it is likely that alternative uracil glycosylase(s) are present in the mitochondria. We conclude that uracil repair is unaffected by inhibition of UDG in the mitochondria.

## Discussion

Cells are exposed to DNA damaging agents generated both as a process of normal physiology as well through extrinsic mutagens. Cells repair damage done to the DNA by a variety of repair mechanisms each specific for the type of DNA damage [23]. Many proteins involved in the repair mechanism are conserved in prokaryotes and eukaryotes. One of the repair mechanisms is the base excision repair pathway that repairs lesions of DNA that involve base modification as well as damage by reactive oxygen species. The enzymes involved in the base excision repair pathway for the repair of the nuclear DNA are well studied [23,24]. Base excision repair involves a DNA glycosylase that cleaves the damaged base by hydrolysis of the glycosidic bond, producing an abasic site. The abasic site generated is then removed by AP endonuclease and the gap is filled by DNA polymerase and then ligated by DNA ligase [23,24]. The first enzyme involved in the base excision repair pathway differs depending upon the lesion introduced in the DNA. Thus uracil DNA glycosylase is specific for misincorporated uracil or deaminated cytosine and would only act on these lesions [11-13]. Oxoguanine DNA glycosylase is specific for 8-oxoguanine and other oxidative species, and 3-methyl adenine glycosylase is specific for alkylated residues [10]. The mitochondrial DNA is subjected to a greater risk of DNA damage due to reactive oxygen species generated as a result of normal physiology of this organelle. The proximity of the mitochondrial DNA to the electron transport chain makes it more vulnerable to the DNA damaging effects of the reactive oxygen species. Therefore, many of the base excision enzymes including UDG have isoforms that are targeted to the mitochondria [9,10].

UDG's role in the nucleus is well established [17]. It is also established that UGI, a PBS2 phage encoded protein when expressed inhibits UDG activity in the nucleus [17,18]. In this paper we investigated whether UDG is the major protein that plays an important role in repairing uracil residues in the mitochondria. In order to address this question, we cloned UGI gene in frame with the mitochondrial localization signal present in the pCMV/myc/mito vector. We isolated stably transfected MCF12A cell lines and measured uracil-DNA repair activity in the mitochondria. We found no difference in DNA repair activity of uracil in mitochondrial extracts. These results were further substantiated by lack of spontaneous mutations in mtDNA in the COXI, COXII and COXIII genes. Similar results were obtained after treating the cells with SIN1 and SNAP that deaminate DNA [22]. Cells expressing UGI also showed no difference in the growth rate suggesting a lack of mitochondrial defect due to UGI inhibition of mitochondrial UDG.

Our results of UGI expression in the mitochondria are different when compared with UGI expression in the nucleus. A previous study has shown that expression of UGI results in inhibition of uracil DNA repair in the nucleus and subsequently mutation in the nuclear DNA [17]. Our results are intriguing and hints to the presence of alternative DNA repair proteins that may repair uracil in mtDNA. Indeed, cells contain several classes of enzymes that can remove uracil residues from DNA and maintain genomic integrity [27]. These include the thymine-DNA glycosylase (TDG), mismatch specific uracil-DNA glycosylase (MUG) and the single-stranded monofunctional uracil-DNA glycosyalse (SMUG1) [27,28]. It is not clear whether any of these proteins are present in the mitochondria and may function as a back up enzyme when UDG is inactivated by UGI.

It is also possible that an extremely low level of mutant mtDNA may be present in the cells expressing UGI in the mitochondria and PCR technique used to identify mutant copies among a heterogeneous population of mtDNA was unable to detect mutant mtDNA molecules. It is also conceivable that targeted UGI is present in a subset of mitochondria and at any given time there is always enough active UDG *in vivo *and in the extract from untargeted mitochondria to carry out the uracil repair activity *in vitro*. However, these possibilities are ruled out because UGI expression did not result in lower level cytochrome C oxidase activity (data not shown).
